# Relationship between knee joint discomfort, self-management behavior, and quality of life in the middle-aged and elderly people in China: A cross-sectional study

**DOI:** 10.3389/fpubh.2022.1029443

**Published:** 2022-12-20

**Authors:** Manman Su, Yang Zhou, Peipei Zhao, Biyun Zeng, Qidi Zhou

**Affiliations:** ^1^Teaching and Research Section of Clinical Nursing, Xiangya Hospital, Central South University, Changsha, Hunan, China; ^2^Operating Room, Xiangya Hospital, Central South University, Changsha, Hunan, China; ^3^Department of Nursing, Xiangya Hospital, Central South University, Changsha, Hunan, China; ^4^Central Intensive Care Unit, Xiangya Hospital, Central South University, Changsha, Hunan, China; ^5^Department of Orthopedics, Xiangya Medical College of Central South University, Changsha, Hunan, China

**Keywords:** knee, self-management, quality of life, middle-aged and elderly, China

## Abstract

**Background:**

The aim of this study was to describe the knee joint discomfort, self-management behavior, and quality of life (QoL) in the middle-aged and elderly people in China and to clarify the relationship between the knee joint discomfort, self-management behavior, and QoL.

**Methods:**

It is a cross-sectional study and in this study, a stratified multistage random sampling method was used to collect data on the three factors such as knee joint discomfort, self-management behavior, and QoL among the middle-aged and elderly people in the Hunan Province. Spearman's correlation analysis was used to test the relationship between the knee joint discomfort, self-management behavior, and QoL.

**Results:**

The results of the present study showed that among them, the prevalence of knee pain was the highest (52.1%), followed by knee weakness (42.5%), numbness (41.8%), cold feeling (40.0%), tenderness (38.3%), and distension feeling (37.5%). Average score of self-management of knee joint discomfort in the middle-aged and elderly people was 2.14 ± 0.67. The level of self-management in each dimension ranged from high to low as emotional management, daily management, symptoms management, and information management. The average scores of physical component summary (PCS) and mental component summary (MCS) were 42.85 ± 5.34 and 43.62 ± 8.43, respectively. The occurrence, frequency, and severity of discomfort symptoms were positively correlated with the symptoms management, daily management, information management, and self-management behaviors, and negatively correlated with the emotional management, PCS and MSC, except for the occurrence of discomfort symptoms (*P* < 0.05).

**Conclusion:**

Knee joint discomfort was prevalent in the middle-aged and elderly people. In addition, they displayed a low level of self-management behavior and poor QoL. The middle-aged and elderly people faced knee discomfort symptoms, the more frequent and severe symptoms, the higher level of symptom management, daily management, and information management, and the lower level of emotional management and QoL.

## Introduction

By the end of 2018, China's elderly population over the age of 60 years had exceeded 240 million, accounting for 17.9% of the total population (1). Globally, joint disease, which becomes more pronounced with age, is the leading cause of disability in the middle-aged and older people (2). Osteoarthritis (OA) is a debilitating common joint disease that affects an estimated 302 million people worldwide (3–5). Large epidemiology studies have found that the hands, knees, and hips are the most commonly affected joints (6). In particular, the knee joints, which bear all the weight of the body, are the most frequently troubled joints (7). With acceleration of the aging process in China, the incidence of knee OA (KOA) is only 2% for those below 45 years of age, 30% for those between 45 and 60 years of age, and 68% for those over 65 years of age (8).

On the basis of recent studies, it is brought to light that KOA is the most common cause of knee discomfort among middle-aged and older adults (9). Knee discomfort is a subjective sensation that may vary significantly depending on the patient and the patient's health condition. In fact, knee discomfort is characterized by severe pain, stiffness, swelling, and loss of normal joint function (10). Previous studies have mainly focused on the severe stage of knee discomfort, such as obvious joint symptoms of KOA and perioperative acute pain of knee replacement (4), while there were a few investigations on the early stage of knee discomfort. In addition, there is a dearth of appropriate multidimensional self-assessment tools to accurately evaluate the characteristics of various symptoms of knee discomfort in the middle-aged and elderly people.

Given the increasing demands for improved health outcomes, self-management behaviors of the middle-aged and elderly people cannot be ignored (11). Evidence shows that adequate self-management behaviors have been linked to preventing or reducing health risks and optimizing the quality of life (QoL) (7). In the present study, older people were more susceptible to knee joint discomfort (12). Despite abundant literature on the effectiveness of self-management programs (13, 14), the paucity of epidemiology data on self-management behaviors and QoL among the middle-aged and elderly people in China appeals for drawing up more population-based surveys. At present, there are no assessment tools or related studies on the self-management of knee joint discomfort in the middle-aged and elderly people in China.

To sum up, the research on the knee joint discomfort of middle-aged and elderly people is still insufficient and many more research works are needed on this subject. The middle-aged and elderly people are the most common groups experiencing knee joint discomfort, and a considerable segment of this population eventually develops clinical diseases. Before the evolution of symptoms, it is important to understand the current status of knee discomfort symptoms and self-management in the middle-aged and elderly population. It is also imperative to provide management recommendations for the prevention or alleviation of knee discomfort. At the same time, it is even necessary to provide a theoretical basis and practical reference for the next exploration of how to effectively implement symptoms management measures for middle-aged and elderly people with knee discomfort.

In this study, we sought to provide overall estimates for the prevalence of knee joint discomfort, self-management behaviors, and QoL among middle-aged and elderly people in China. “The middle-aged and elderly” refers to individuals aged 45 years and above. Additionally, the relationship between knee joint discomfort, self-management behaviors, and QoL was analyzed, hoping to fill the vacancy and provide evidence for policymakers.

## Materials and methods

### Study design

A cross-sectional study design was adopted from 15 January 2020 to 31 May 2020. The study was conducted in two cities of “Changsha” and “Zhangjiajie” in the Hunan Province, China.

### Setting

Two cities (states) were randomly selected from among 14 cities and states in the Hunan Province, namely Changsha City and Zhangjiajie City. The participants had lived in the city for at least 6 months.

### Participants

#### Inclusion and exclusion criteria

The inclusion criteria were as follows: (1) Age of the participant should be ≥45; (2) the participant should be a community resident in the Hunan Province of China for >6 months; (3) the participant should possess normal communication skills; and (4) the participant should agree to take part on a voluntary basis and should have signed an informed consent form.

The exclusion criteria were as follows: (1) The participant had a history of knee surgery and (2) the participant expressed the inability to participate or is unwilling to cooperate with the investigation.

#### Sample size calculation

The minimum sample size was calculated by the equation *n* = *t*^2^*pq/d*^2^, which was equal to 7,968. Considering a dropout rate of 20%, the target sample size was 9,562. Eventually, 10,000 individuals who met the inclusion criteria were included and 9,993 questionnaires were returned. In this study, 9,640 valid questionnaires were collected.

#### Sampling procedure

**Figure 1:**
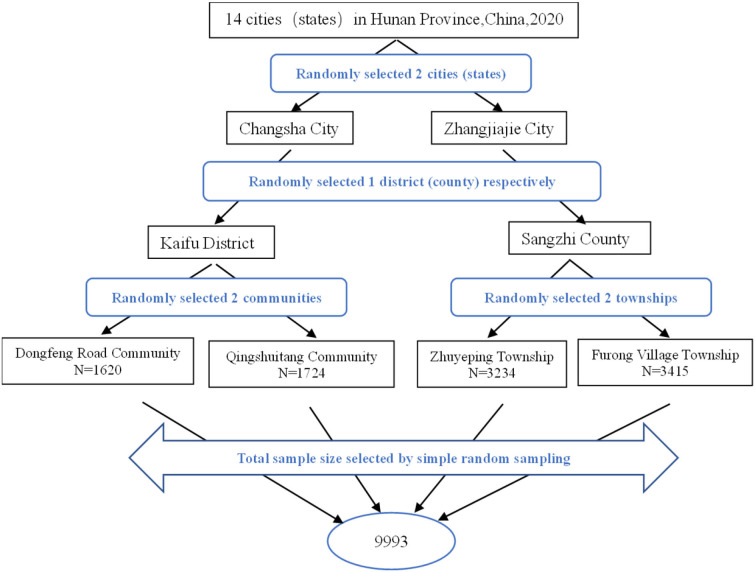
Flowchart for screening the subjects (*N* = 9,993).

The stratified multistage random sampling method was used to collect samples. First, two cities (states), namely Changsha City and Zhangjiajie City, were randomly selected from among the 14 cities (states) in the Hunan Province; then, one district (county), respectively, namely Kaifu District and Sangzhi County, was randomly selected from each of the selected cities (states) by further stratification; finally, two communities, namely Dongfeng Road Community (*N* = 1,620) and Qingshuitang Community (*N* = 1,724), and two townships, namely Zhuyeping Township (*N* = 3,234) and Furong Village Township (*N* = 3,415), were randomly selected from among the selected districts (counties). The total sample size selected by simple random sampling stands at *N* = 9,993. Figure 1 shows the flowchart for screening the subjects in this study.

### Instruments

The survey tools consist of four parts, including General Information Questionnaire, Self-Rating Scale for Knee Joint Unpleasant Symptoms, Self-management Behavior Scale for Knee Joint Discomfort, and the 12-item Short Form Health Survey.

#### Demographic characteristics

The questionnaire was formulated based on demographic characteristics of the middle-aged and elderly people, including age, gender, BMI, ethnicity, educational level, marital status, dwelling state, source of finance, monthly income, residence, smoking, and alcohol drinking.

#### Self-Rating Scale for Knee Joint Unpleasant Symptoms

Self-Rating Scale for Knee Joint Unpleasant Symptoms in the middle-aged and elderly people was used to measure the knee joint discomfort. It was developed by our research team in 2020 (15), based on the middle-range theory of unpleasant symptoms (16). As the study was conducted in China, the scale was designed in Chinese and translated into English as given in the present manuscript. This scale consists of two dimensions, subjective symptoms (items 1–14) and self-examination signs (items 15–21), with 21 items. Subjective symptoms are evaluated from three aspects: incidence, frequency, and severity. In addition, self-examination signs are assessed from two sides: incidence and severity. The frequency is divided into four grades: rare, sometimes, frequent, and continuous, from 1 to 4, respectively. The severity is divided into mild, moderate, severe, and unbearable, with four grades ranging from 1 to 4. The content validity index (CVI) for each item ranges from 0.777 to 1.000, the average CVI for all items is 0.891, and the total scale has a Cronbach's alpha of 0.949, the half-reliability is 0.888, and the test-retest reliability is 0.914 (15).

#### Self-management Behavior Scale for Knee Joint Discomfort

Self-management behavior for knee joint discomfort was measured using the Self-management Behavior Scale for Knee Joint Discomfort developed by Peipei et al. (17) for the middle-aged and elderly. This scale consists of 27 items in four dimensions: symptoms management (ten items), daily management (nine items), emotion management (four items), and information management (four items). Each item is scored from 1 to 5 points (1 = none, 2 = few, 3 = sometimes, 4 = often, and 5 = always), and the total scores range from 27 to 135. A higher total score indicates better self-management skills. The CVI of each item ranges from 0.833 to 1.000, and the average CVI of all items is 0.964. The Cronbach's alpha of the total scale is 0.946, the split-half reliability is 0.894, and the test-retest reliability is 0.876 (17).

#### The 12-item Short Form Health Survey

The QoL was assessed using the 12-item Short Form Health Survey (SF-12) simplified from SF-36 by the Boston Institute of Health Education (18). It involves 12 items in eight dimensions: general health (GH), physical functioning (PF), role-physical (RP), bodily pain (BP), vitality (VT), social functioning (SF), role-emotional (RE), and mental health (MH). Physical component summary (PCS) can be obtained by calculating the score of GH, PF, RP, and BP; SF, RE, MH, and VT add up to mental component summary (MCS). The score ranges from 0 to 100 (0 = worst, 100 = best), and a score equal to or >50 is normal. And the higher the score, the better the subjective feeling of the respondents and the better the quality of life. The total scale of Cronbach's alpha is 0.760–0.860, and Cronbach's alpha of each dimension is 0.700–0.778 (19).

### Data collection procedure

Before conducting the study, the study proposal and questionnaire were reviewed and approved by the Medical Ethics Committee of the Xiangya Hospital of Central South University. Members of the research team who received unified training visited different communities or townships. The training was provided to each local collector on methodology, survey tools, data collection, ethical issues, and how to supervise the data collection process. The purpose, benefits, and risks of participation in the research were also explained to potential participants. Participants were informed that their participation was voluntary and they had the right to withdraw at any time. After obtaining the consent of the participants, the questionnaire was answered under one-on-one guidance. Items difficult to understand were explained by the researcher to ensure consistency in the interpretation of each item. Each questionnaire took about 15–20 min to fill. All questionnaires were completed anonymously and the answers of participants did not involve any personal assessment. The information provided by the participants was confidential and the survey would not cause any harm to the participants. The completed questionnaires were assured of completeness and consistency during the survey period. The collected data were entered into the database and rechecked. Initially, 9,993 middle-aged and elderly people consented to participate in the study and received the questionnaires. Of these, 353 were ultimately excluded from the analysis due to incomplete or missing data. In total, 9,640 questionnaires were eligible for data analysis in the study, resulting in a response rate of 96.5%.

### Statistical analysis

In this research, IBM SPSS Statistics 23.0 was used for statistical analysis. Descriptive statistics were used for presenting demographic characteristics, knee joint discomfort, self-management behavior, and quality of life in the middle-aged and elderly. Categorical variables are expressed as absolute frequencies (percentages), while continuous variables are expressed as mean ± SD (standard deviation) or median (interquartile range, IQR), as appropriate. The Kolmogorov-Smirnov test was used to check the normal distribution of continuous data. Spearman's correlation analysis was used to test the relationship between the knee joint discomfort, self-management behavior, and QoL. All *P*-values < 0.05 were considered statistically significant.

## Results

### Demographic characteristics of the middle-aged and elderly

The demographic characteristics of the middle-aged and elderly people are shown in Table 1. The average age of the middle-aged and elderly people was 58.99 ± 12.00 years, and an overwhelming number of them were female (52.7%). The BMI was mostly <24 kg/m^2^ (67.2%) and as regards ethnicity of the study participants, the Han Chinese were predominant and occupied 51.6% of the total number of study participants, while the other ethnic minorities occupied 48.4%. As regards the educational level of the participants, the elementary school and below level of education accounted for 39.2%. The great majority of them were married (86.7%) and lived with family members (87.6%). The source of finance mainly came from salary earnings (57.9%) and the average family income per month stood at 1,000–3,000 RMB (41.9%). As regards residence, most of the people lived in the countryside (69.8%), while the number of urban dwellers was comparatively less. A portion of the population had smoking (23.1%) and alcohol drinking (35.8%) habits. While the majority of the participants were non-smokers (76.9%) and non-drinkers (64.2%), the number of drinkers stood at 35.8%.

**Table 1:** Demographic characteristics of the middle-aged and elderly people (*n* = 9,640).

**Variable**	***n* (%)**	**Mean ±SD**
**Age (years)**		
45–50	3,485 (36.2)	58.99 ± 12.00
51–60	2,038 (21.1)	
61–70	2,312 (24.0)	
71–99	1,805 (18.7)	
**Gender**		
Male	4,559 (47.3)	
Female	5,081 (52.7)	
**BMI (kg/m** ^2^ **)**		
<24	6,480 (67.2)	23.25 ± 5.96
24–27.99	2,062 (21.4)	
≥28	1,098 (11.4)	
**Ethnicity**		
Han Chinese	4,979 (51.6)	
Ethnic minorities	4,661 (48.4)	
**Educational level**		
Elementary school and below	3,775 (39.2)	
Junior high school	3,256 (33.8)	
Technical/high school	1,216 (12.6)	
Diploma/bachelor degree	1,247 (12.9)	
Master degree and above	146 (1.5)	
**Marital status**		
Unmarried	348 (3.6)	
Married	8,355 (86.7)	
Divorced	211 (2.2)	
Widowed	26 (7.5)	
**Dwelling state**		
Solitary	773 (8.0)	
Living with family	8,442 (87.6)	
Living in a nursing home	155 (1.6)	
Others	270 (2.8)	
**Source of finance**		
Salary	5,582 (57.9)	
Others	4,058 (42.1)	
**Monthly family income (RMB)**		
<1,000	2,248 (23.3)	
1,000–3,000	4,041 (41.9)	
3,001–5,000	2,036 (21.1)	
5,001–10,000	945 (9.8)	
>10,000	370 (3.8)	
**Residence**		
Urban	2,915 (30.2)	
Countryside	6,725 (69.8)	
**Smoking**		
Smoker	2,227 (23.1)	
Non-smoker	7,414 (76.9)	
**Alcohol drinking**		
Drinker	3,453 (35.8)	
Non-drinker	6,187 (64.2)	

BMI, body mass index; BMI, weight (kg)/height^2^ (m).

### Knee joint discomfort of the middle-aged and elderly people

As shown in Table 2, the prevalence of knee pain was the highest (52.1%), followed by knee weakness (42.5%), numbness (41.8%), cold feeling (40.0%), tenderness (38.3%), and distension feeling (37.5%). Knee pain was the most frequent and continuous subjective symptom (9.5%) and 5.9% of the middle-aged experienced severe and unbearable pain.

**Table 2:** Prevalence of knee joint discomfort among the middle-aged and elderly people (*n* = 9,640).

**Knee joint discomfort**	***n* (%)**	**Frequency was “frequent and continuous” (*n*, %)**	**Severity was “severe and unbearable” (*n*, %)**	**Frequency (mean ±SD)**	**Severity (mean ±SD)**
**Subjective symptoms**					
3. Pain	5,022 (52.1)	916 (9.5)	569 (5.9)	1.94 ± 0.74	1.59 ± 0.75
13. Weakness	4,096 (42.5)	577 (6.0)	328 (3.4)	1.78 ± 0.75	1.45 ± 0.68
2. Numbness	4,032 (41.8)	542 (5.6)	269 (2.8)	1.82 ± 0.71	1.43 ± 0.65
1. Cold feeling	3,854 (40.0)	433 (4.5)	196 (2.3)	1.78 ± 0.70	1.40 ± 0.64
5. Tenderness	3,696 (38.3)	610 (6.3)	346 (3.6)	1.86 ± 0.75	1.50 ± 0.72
4. Distension feeling	3,616 (37.5)	465 (4.8)	304 (3.2)	1.79 ± 0.71	1.47 ± 0.69
14. Muscle cramps	3,509 (36.4)	512 (5.4)	351 (3.7)	1.80 ± 0.75	1.50 ± 0.76
6. Stiffness	3,382 (35.1)	503 (5.3)	263 (2.8)	1.80 ± 0.75	1.45 ± 0.68
12. Heaviness	3,220 (33.4)	464 (4.8)	251 (2.6)	1.80 ± 0.75	1.46 ± 0.68
8. Snapping	3,197 (33.2)	424 (4.4)	193 (2.0)	1.75 ± 0.75	1.39 ± 0.64
7. Friction feeling	3,150 (32.7)	455 (4.7)	215 (2.2)	1.79 ± 0.74	1.42 ± 0.65
9. Instability	2,956 (30.7)	399 (4.1)	254 (2.7)	1.75 ± 0.75	1.46 ± 0.70
10. Noose feeling	2,365 (24.5)	278 (2.9)	224 (2.4)	1.69 ± 0.75	1.44 ± 0.71
11. Skin temperature arise	2,320 (24.1)	254 (2.6)	143 (1.5)	1.69 ± 0.73	1.39 ± 0.65
**Self-examination signs**					
15. Full knee extension	1,663 (17.3)	/	156 (1.7)	/	1.52 ± 0.74
16. Full knee flexion	1,486 (15.4)	/	178 (1.8)	/	1.59 ± 0.77
17. Claudication	1,455 (15.1)	/	202 (2.1)	/	1.63 ± 0.78
18. Swelling	1,440 (14.9)	/	186 (2.0)	/	1.61 ± 0.75
20. Protuberance	1,192 (12.4)	/	143 (1.5)	/	1.59 ± 0.77
19. Deformity	1,163 (12.1)	/	127 (1.3)	/	1.53 ± 0.75
21. Muscle atrophy	1,104 (11.5)	/	149 (1.6)	/	1.59 ± 0.78

### Knee joint discomfort self-management and quality of life

As shown in Table 3, the average score of self-management of knee joint discomfort in the middle-aged and elderly people was 2.14 ± 0.67. The emotion management was the highest, followed by daily management, symptoms management, and information management. The average scores of PCS and MCS were 42.85 ± 5.34 and 43.62 ± 8.43, respectively.

**Table 3:** Mean, standard deviation, minimum, and maximum of knee joint discomfort self-management and quality of life (*n* = 9,640).

	**Mean**	**SD**	**Min**	**Max**
Knee joint discomfort self-management	2.14	0.67	1.15	5.00
Symptoms management	1.96	0.81	1.00	5.00
Daily management	2.05	0.90	1.00	5.00
Emotion management	3.12	0.53	1.00	5.00
Information management	1.83	0.92	1.00	5.00
Quality of life	/	/	/	/
PCS	42.85	5.34	17.46	62.68
MCS	43.62	8.43	9.99	68.53

### Correlations of major study variables

The correlations of major study variables among middle-aged and elderly people are shown in Table 4. The results of the study showed that the occurrence, frequency, and severity of discomfort symptoms were positively correlated with symptoms management, daily management, information management, and self-management behaviors, and negatively correlated with emotional management, PCS and MSC, except for the occurrence of discomfort symptoms (*P* < 0.05).

**Table 4:** Correlations of major study variables (*n* = 9,640).

**Variable**	**Symptoms management**	**Daily management**	**Emotion management**	**Information management**	**Self-management**	**PCS**	**MCS**
Occurrence	0.465^**^	0.340^**^	0.013^*^	0.296^**^	0.409^**^	−0.324^*^	−0.300^*^
Frequency	0.596^**^	0.402^**^	−0.089^**^	0.372^**^	0.499^**^	−0.578^**^	−0.571^**^
Severity	0.598^**^	0.400^**^	−0.100^**^	0.377^**^	0.498^**^	−0.382^**^	−0.390^**^

*P < 0.05.

**P < 0.01 (2-tails).

## Discussion

### Knee joint discomfort was prevalent in the middle-aged and elderly people

To our knowledge, this is the first quantitative study to provide the overall prevalence of knee joint discomfort in the middle-aged and elderly people in China. For knee joint discomfort, there are not enough articles available to discuss, except the main focused symptom of “pain.” Previous literature related to the word “discomfort” was reviewed; however, questionnaires are commonly used to study “pain” rather than “discomfort” in most of the publications (20). Actually, pain is just one of the symptoms of knee joint discomfort. In this study, more than half (52.1%) of 9,640 middle-aged and elderly populations in Hunan province had pain. Furthermore, the frequency that ranged from frequent to almost continuous was 9.5%, and the severity that ranged from severe to unbearable was 5.9%, indicating that more than half of the middle-aged and elderly suffered from knee joint pain, and some of them had even severe symptoms of knee joint pain. We explored with other symptoms, not limited to the highest incidences of “pain.” They included knee weakness (42.5%), numbness (41.8%), cold feeling (40.0%), tenderness (38.3%), and distension feeling (37.5%). This suggests that middle-aged and older adults often experience multiple knee discomfort symptoms. Healthcare workers and middle-aged and elderly people themselves should pay more attention to the health care of the knee joint, not only in the management of its symptoms and health education, but also in gradually cultivating the awareness of nutrition and health care related to the knee joint.

Additionally, we found that some symptoms had a high incidence or frequency, but were not the most serious. For example, “Muscle cramps” vs. “Weakness” symptom. This inconsistency between different dimensions illustrates the importance of a comprehensive assessment of knee discomfort symptoms. Based on an overall assessment of knee discomfort symptoms in the middle-aged and older adults, interventions targeting high severity and frequency of symptoms are necessary for a better symptom management.

### Middle-aged and elderly people had a low level of self-management behavior

In our research, we found that the average score of self-management of knee joint discomfort in the middle-aged and elderly people was at a low level (2.14 ± 0.67). The literature describing the self-management behavior of knee joint discomfort among elderly people in China is scarce. In terms of dimension, we found that the elderly strived for an optimal emotion management. However, their coping skills in daily management, especially information management and symptoms management, were lacking. Patients in a study by Miller et al. (21) reported that they attempted to adjust their lifestyle to reduce knee joint symptoms. Conceivably, those with knee discomfort seek information on their own initiative, using a variety of sources, such as multimedia platforms and peers, to gain control of their condition (11). Therefore, in order to increase their knowledge on how to self-manage problems to improve their QoL (22), health professionals should ensure that the elderly with knee joint discomfort receive individualized self-management plans and lifestyle advice. Our study may provide some insight into the design and implementation of future self-management interventions.

### Middle-aged and elderly people appeared to have poor QoL

This study demonstrated that the QoL of middle-aged and elderly people was very poor, with average scores of PCS and MCS (42.85 ± 5.34) and (43.62 ± 8.43), respectively, which were significantly lower than those given in the study by Hu et al. (23) (68.57 ± 24.21) and (74.17 ± 22.96) for patients with hypertension in the rural areas of Lianyungang City. Additionally, the QoL in our study appears to be higher than the level of elderly population in the study by Palo et al. (24) on knee OA patients in India and the study by Choojaturo et al. (22) in Thailand. In large epidemiology studies, OA has been reported as the major common cause affecting function and QoL in older adults worldwide (6). Although many health policies and countermeasures have been adopted, the QoL of elderly people remains very disagreeable in both developed and developing nations (25, 26). In our research, 69.3% of the elderly lived in rural areas and the majority of them had a low knowledge level and socioeconomic status (SES). In reality, these factors may in some way become barriers to adequate access to health services. There is already evidence to suggest that individuals with lower SES have poorer QoL (27). These situations raise important questions about what factors, if any, can improve healthcare accessibility and QoL for elderly people.

### Relationship between knee joint discomfort, self-management behavior, and QoL

This study indicated that middle-aged and elderly people had knee discomfort symptoms, the more frequent and severe the symptoms, the higher level of symptom management, daily management, and information management, and the lower level of emotional management and QoL. The correlation between the variables suggests the importance and necessity of knee self-management in the middle-aged and elderly people. This fact also indirectly indicates that different degrees of knee discomfort symptoms may be related to self-management behaviors, and the results are also consistent with the interpretation of clinical phenomena. The correlation between different levels of knee discomfort symptoms and self-management behaviors may also be explored in later studies.

### Strengths and limitations

Our study has several strengths. First, although previous studies have been conducted on knee OA, to the best of our knowledge, this is the first study to look at the prevalence of knee joint discomfort and the level of self-management behaviors and QoL among the middle-aged and elderly people in the Chinese population. As the aging process accelerates and the life expectancy increases, there is a need to study China's middle-aged and older populations because of the significant differences in culture and healthcare system compared to other countries. Second, the scales used in this study were specifically designed to measure the symptoms and self-management of people with knee discomfort, which contributed to the validity and reliability of the results. Moreover, the validated scale can be used as a theoretical basis for developing and observing the effects of self-management programs in the future. Third, the collected data were analyzed using reliable statistical methods, and the results may provide theoretical support for future knee management interventions.

Some limitations need to be addressed. First, the study was just conducted in the Hunan province of China, which might limit the generalizability of the results. Further research is needed to increase the sample size and extend the finding to populations in other parts of China. Second, recall bias may exist due to the long span of time. Last, our study is a cross-sectional investigation; cross-sectional study designs may not be able to determine the causal relationship between the variables of interest.

## Conclusion

Knee joint discomfort was prevalent in the middle-aged and elderly people. In addition, they have a low level of self-management behavior and poor QoL. This study indicated that the middle-aged and elderly people had knee discomfort symptoms, the more frequent and severe the symptoms, the higher level of symptom management, daily management and information management, and the lower level of emotional management and QoL. It is necessary to conduct a further study, such as on in-depth coping strategies used by middle-aged and elderly people themselves experiencing knee joint discomfort, to increase their self-management skills and improve their quality of life.

## Data availability statement

The raw data supporting the conclusions of this article will be made available by the authors, without undue reservation.

## Ethics statement

The studies involving human participants were reviewed and approved by the Research Ethics Board of the Xiangya Hospital of Central South University. The participants provided their written informed consent to participate in this study. Written informed consent was obtained from the individual(s) for the publication of any potentially identifiable images or data included in this article.

## Author contributions

MS designed the study, participated in the data processing and statistical analysis, and wrote the initial draft of the manuscript. PZ, BZ, and QZ participated in the designing of the study, administration of the questionnaire, and discussed the analytical results. YZ provided important feedback on the manuscript, participated in the designing of the study, and provided important feedback on the manuscript. All authors read and approved the final manuscript.
